# Comparison between Conduction and Convection Effects on Self-Heating in Doped Microcantilevers

**DOI:** 10.3390/s120201758

**Published:** 2012-02-09

**Authors:** Mohd Zahid Ansari, Chongdu Cho

**Affiliations:** Department of Mechanical Engineering, Inha University, 253 Yonghyun-dong, Nam-Ku, Incheon 402-751, Korea; E-Mail: ansari.zahid@hotmail.com

**Keywords:** microcantilever, self-heating, biosensors, hotplates, piezoresistivity, electrical resistivity, bimetallic effect

## Abstract

The present study investigates the effects of thermal conduction and convection on self-heating temperatures and bimetallic deflections produced in doped microcantilever sensors. These cantilevers are commonly used as sensors and actuators in microsystems. The cantilever is a monolith, multi-layer structure with a thin U-shaped element inside. The cantilever substrate is made of silicon and silicon dioxide, respectively, and the element is p-doped silicon. A numerical analysis package (ANSYS) is used to study the effect of cantilever substrate material, element width, applied voltage and the operating environments on cantilever characteristics. The numerical results for temperature are compared against their analytical models. Results indicate the numerical results are accurate within 6% of analytical, and Si/Si cantilevers are more suitable for biosensors and AFM, whereas, Si/SiO_2_ are for hotplates and actuators applications.

## 1. Introduction

Doped microcantilevers have found applications in numerous novel microsystems such as AFM [1], data storage [2], biomolecular force sensors [3], microheaters [4], biosensors [5], biochemical sensors [6], DNA sequencing [7] and immunosensors [8]. A detailed review on the theory and applications of piezoresistance in microsystems can be found in [9]. To achieve a particular functionality, the microcantilevers, normally made of a semiconductor material, are doped with a suitable dopant in a predetermined implantation dose concentration. The doping creates a very thin electrical element in the cantilever. The element can be a simple electrical resistor or a piezoresistor. For sensor applications, the element is normally made piezoresistive, whereas, for actuators the element is a resistor. The cantilevers can also be made self-sensing and self-actuating [10,11]. Piezoresistivity is the change in bulk electrical resistivity of a material caused by an applied mechanical stress or strain. In sensing the cantilever deflections and strains are measured indirectly by using the resistance change, whereas, in actuation the deflections are produced by using the bimorph bending in the cantilever. In all these applications, an electrical current is made to pass through the element, which produces self-heating in the cantilever. Self-heating is an energy dissipation phenomenon that irreversibly converts electrical energy into thermal energy. This is commonly observed in electrical current carrying conductors. Most of the thermal energy is generated due to the loss of kinetic energy of the current carrying electrons by collisions among themselves and with the lattice atoms. The amount of heat generated is directly proportional to the electrical resistivity of the conductor and the square of the magnitude of the electrical current passing through it. The present study will discuss the self-heating effects associated with biosensor, AFM, hotplate and actuator applications of doped microcantilevers.

Doped microcantilevers with piezoresistor elements are particularly attractive for biosensors and applications because of their label-free, rapid, real-time and integrated-readout features. These sensors measure indirectly the variation in the surface stress produced by the analyte-receptor binding to determine the analyte and its concentration. The surface stress variations produce deflections and strains in the cantilever, and change the electrical resistance of the element because of piezoresistivity. The self-heating associated with piezoresistive microcantilever biosensors is, however, a major source of noise. The increase in cantilever temperature changes the electrical properties of the element because of temperature coefficients of resistivity (TCR) and piezoresistivity (TCP) and produce bimorph bending in cantilever as well. These thermal effects greatly influence the sensitivity and resolution of the biosensor. Therefore, self-heating in the cantilevers should be minimised to improve the accuracy of the biosensor. The integrated-readout capability offered by piezoresistive microcantilevers is also very useful in AFM. Since the principle of deflection-readout arrangement in AFM is similar to the biosensors discussed above, the sources of noise in the two cases are also similar. Self-heating effects, however, can also be used to practical applications in form of hotplates and actuators. In these applications, the element is generally an electrical resistor. However, for self-sensing ability the resistor can be replaced by a piezoresistor. Microheaters and hotplates are used to generate and maintain the required operating temperatures to provide a controlled microenvironment to study various physical, chemical and biological processes. Other useful application of self-sensing is in form of actuators. The bimorph bending in the cantilever can be used to actuate a mechanism with sensing features by using a feedback control system. In all the above applications, accurate prediction of the temperature and its distribution in the cantilever is critical for precise functioning of the sensor or the actuator.

The objective of the present work was to study the effect of changes in the operating medium on the temperature and deflections produced in doped microcantilevers. The cantilevers are assumed to be used in gaseous and liquid environments. Cantilevers in two configurations are studied. In the first case, both element and the cantilever are made is silicon (*i.e*., Si/Si). In the second case, the element is silicon but the cantilever substrate is silicon dioxide (*i.e*., Si/SiO_2_). The element is U-shaped and is made of p-doped silicon. The cantilevers are subjected to different applied voltages and element widths. A numerical analysis package (ANSYS Multiphysics v.12) was used to determine the temperature and deflection characteristics of the cantilevers. Finally, the self-heating associated with doped microcantilevers used in different applications is discussed.

## 2. Theory and Simulation

**Figure 1.**
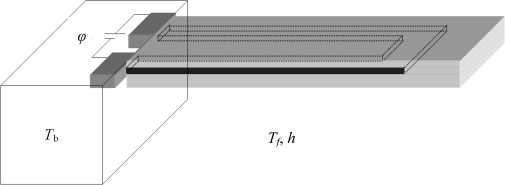
Schematic design of a doped microcantilever with U-shaped element.

The thermal energy generated in the element by self-heating is mostly diffused by conduction within the cantilever and the cantilever base to achieve the thermal equilibrium. In addition, heat is also dissipated into the ambient environment by convection and radiation modes of heat transfer. In general, the radiation losses are insignificant compared to convection. Thus, only the conduction and convections modes are generally significant. The conduction and convection are competing effects, because conduction tries to spread the heat within the cantilevers but the convection removes the heat from the cantilever. The relative contribution of convection-to-conduction effect can be predicted by Nusselt number given as *Nu* = *h x*/*k*, where *h* is convective coefficient of heat transfer, *x* is characteristic length and *k* is thermal conductivity. Thus, if the cantilever is operated in high *h* environment *Nu* will be high, and the convective heat loss will be significant. Typical values of *h* for free convection range 2–25 for gases and 50–1,000 for liquids, where as those for forced convection range 25–250 and 100–20,000, respectively [12]. In the present study, we assume the cantilevers are operated in low-pressure gaseous environment with *h* = 0 and in liquid environment with *h* = 1,000 W/m^2^·°C, respectively. Figure 1 shows the schematic design of a typical doped microcantilever with a U-shaped element. The element can be piezoresistor or resistor depending on the application. The element and the electrical contacts are made by doping. The applied voltage across the contacts causes electrical current flow in the element. The current passing through the element, which has a definite value of electrical resistance, produces self-heating in the cantilever.

If convection heat losses are negligible (*i.e.*, *h* = 0), the temperature distribution along the length in the doped microcantilever, can be given as [13]:

(1) $$T_{c}(x)=\frac{T_{b}}{2}\left[e^{\sqrt{C_{1}}x}+e^{-\sqrt{C_{1}}x}\right]+\frac{C_{2}x^{2}}{2}$$

and the maximum temperature as:

(2) $$T_{c,\text{max}}=\frac{T_{b}}{2}\left[e^{\sqrt{C_{1}}L}+e^{-\sqrt{C_{1}}L}\right]+\frac{C_{2}L^{2}}{2}$$

where:

$$C_{1}=\frac{\eta \varphi^{2}A_{\mathrm{pzr}}}{\rho_{e,0}k_{\mathrm{eff}}L_{\mathrm{pzr}}V}\;\text{and}\;C_{2}=\frac{(1-\eta T_{0})\varphi^{2}A_{\mathrm{pzr}}}{\rho_{e,0}k_{\mathrm{eff}}L_{\mathrm{pzr}}V}$$

where *T*_b_ is cantilever base temperature, *φ* is applied voltage, *A*_pzr_ is cross-sectional area of the element, *L*_pzr_ is total length of the element and *V* is volume of the cantilever containing the element, *η* is TCR and *ρ*_e,0_ is the resistivity defined at reference temperature *T_0_*. *k*_eff_ is the effective thermal conductivity of the layered microcantilever structure, given as *k*_eff_ = ∑ *m*_i_*n*_i_*k*_i_, where *m* and *n* are the width and thickness ratios of the layer-to-cantilever for the constituent materials. A modified form of Equation (1) applicable to microcantilevers with a short element can be found in [14]. Equation (1) is valid for the condition that convection losses are negligible and the heat is diffused into the entire cantilever by conduction. However, if the cantilever is operated in liquid environments the effect of *h* on temperatures produced can be significant and the inclusion of *h* in above relations becomes necessary. In such a case, the heat transfer problem transforms from conduction-only to the conduction-convection model. The modified form of Equation (1) including the convection heat loss can be given as [15]:

(3) $$T_{h}(x)=T_{f}+\left[T_{c}(x)-T_{f}\right]\frac{\text{cosh}\;\beta (L-x)}{\text{cosh}\;\beta L},\;\;\;0<x\leq L,\;\;\beta =\sqrt{\mathrm{hP}/k_{\text{eff}\;}A_{c}}$$

and the maximum temperature as:

(4) $$T_{h,\text{max}}=T_{f}+\left[T_{c,\text{max}}-T_{f}\right]\frac{1}{\text{cosh}\;\beta L}$$

where *T*_f_ and *h* are the temperature and the heat transfer coefficient of the ambient fluid and *A*_c_ and *P* are the cross-sectional area of the cantilever and its perimeter.

The simulations involved cantilevers models with different applied voltages, piezoresistor widths, cantilever materials and operating environments. The applied voltages were increased from 5 to 10 V. The width was changed as 15 μm, 30 μm, and 45 μm. The total cantilever size was 200 μm × 100 μm × 0.75 μm. The element length and thickness was 180 μm and 0.1 μm, respectively. The top-down thickness of the cantilever layers are 0.05 μm (Au), 0.1 μm (SiO_2_), 0.1 μm (Si element) and 0.5 μm (SiO_2_). The effect of piezoresistor length on self-heating in biosensors can be found in [14,15]. The cantilevers material was changed from silicon and silicon dioxide. The element was, however, made of p-doped silicon in both the cases. The operating environments was changed from *h* = 0 to *h* = 1,000 W/m^2^·°C. Thus, in total 72 cases were studied in the numerical analysis. The simulations used the commercial ANSYS Multiphysics v.12 finite element analysis software to numerically study the temperature distribution and thermal deflection in the microcantilevers due to self-heating. About 100,000 coupled field 8-node scalar SOLID5 elements used in 3-D analysis of each case and were solved under steady-state condition. Table 1 presents the material properties of the cantilever.

## 3. Results and Discussion

**Figure 2.**
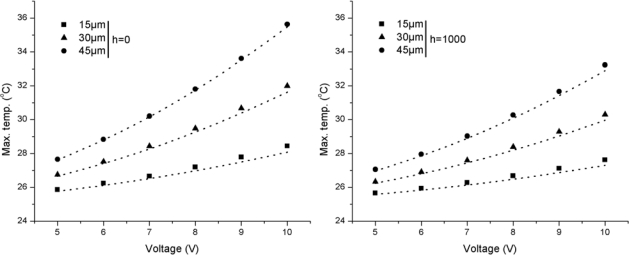
Comparison between simulation and analytical results for maximum temperature in Si/Si cantilevers for. Symbols represent simulation results.

Figure 2 presents the comparison between simulation and analytical results for Si/Si cantilevers operated at *h* = 0 and *h* = 1,000 W/m^2^·°C. The element and the substrate were made of Si. The analytical results were determined from Equations (2) and (4), respectively, for the conduction-only case (*i.e.*, *h* = 0) and the conduction-convection case (*i.e.*, *h* = 1,000 W/m^2^·°C). The element width was 15 μm, 30 μm, and 45 μm, and the applied voltage was increased from 5 to 10 V. It can be seen in the figure that the simulation results match well to the analytical results. The maximum deviation between the two results for *h* = 0 and *h* = 1,000 W/m^2^·°C are 1.28%, 1.16% and 0.45%, and 1.14%, 1.11% and 1.01% for the widths 15 μm, 30 μm and 45 μm respectively. The change in operating medium from *h* = 0 to *h* = 1,000 W/m^2^·°C decreased the maximum temperatures from 28.44, 32.01 and 35.63 °C to 27.62, 30.30 and 33.23 °C for the widths 15 μm, 30 μm and 45 μm, respectively. The reduction in temperatures is understandable because the high value of convective coefficient of heat transfer results in high heat loss from the cantilever.

**Figure 3.**
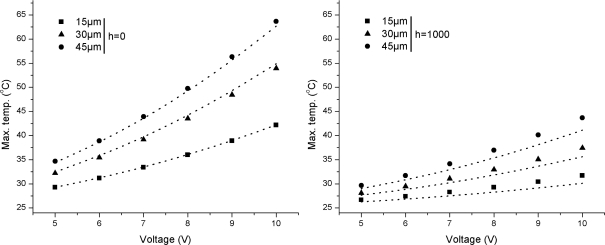
Comparisons between simulation and analytical results for maximum temperatures in Si/SiO_2_ cantilevers. Symbols represent simulation results.

The comparisons between simulation and analytical results for Si/SiO_2_ cantilevers operated at *h* = 0 and *h* = 1,000 W/m^2^·°C are shown in Figure 3. In this case, the element material was Si but the substrate was SiO_2_. The results were determined from Equations (2) and (4), as before. The simulation results show good accord with the analytical results. The maximum absolute deviation between the two results for *h* = 0 and *h* = 1,000 W/m^2^·°C are 0.09%, 1.76% and 1.52%, and 5.02%, 4.76% and 5.88%, respectively for the three widths. The deviation values observed in case of Si/SiO_2_ cantilevers are higher than Si/Si especially in case of *h* = 1,000 W/m^2^·°C. It can also be observed in Figure 3 that the temperature decrease caused by the change in operating medium is more influential in case of Si/SiO_2_ cantilevers. The change from *h* = 0 to *h* = 1,000 W/m^2^·°C decreased the maximum temperatures from 42.17, 53.94 and 63.66 °C to 31.70, 37.41 and 43.68 °C for the widths 15 μm, 30 μm and 45 μm, respectively.

**Figure 4.**
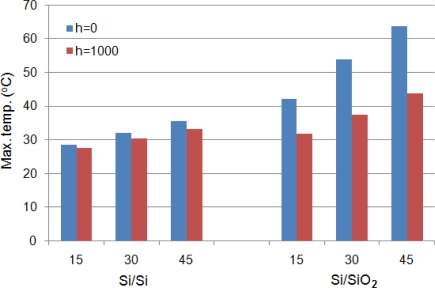
Comparison between maximum temperatures in Si/Si and Si/SiO_2_ cantilevers at 10 V.

Figure 4 shows the comparison between maximum temperatures observed in Si/Si and Si/SiO_2_ cantilevers with different element widths for *h* = 0 and *h* = 1,000 W/m^2^·°C operated at 10V. It is obvious in the figure that the change in cantilever substrate material from Si to SiO_2_ produced higher temperatures. This can be explained by Equation (1), which states the temperature produced is inversely related to the thermal conductivity of the cantilever material. Since the thermal conductivity of Si is more than 100 times SiO_2_ (Table 1), the temperature increase will be lower in case of Si cantilevers. The change in operating medium from *h* = 0 to *h* = 1,000 W/m^2^·°C results in temperature decrease in both cantilevers. The decrease is, however, more pronounced in Si/SiO_2_ cantilevers. This can be explained by the relatively higher temperature gradient existing for convective heat loss in Si/SiO_2_ cantilevers. In other words, high temperatures also produce relatively high temperature decrease during convection heat loss.

**Figure 5.**
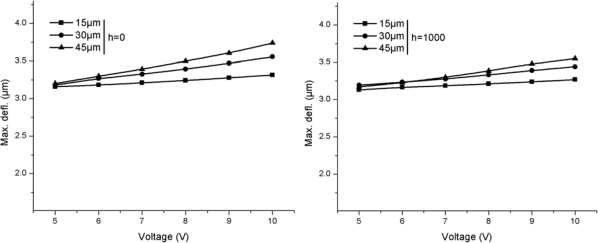
Maximum deflections in Si/Si cantilevers.

Figure 5 presents the maximum deflections produced in Si/Si cantilevers operated at *h* = 0 to *h* = 1,000 W/m^2^·°C. The deflections are produced because of the bimorph bending action in the cantilevers. The maximum deflections for *h* = 0 are 3.32 μm, 3.56 μm and 3.74 μm, and for *h* = 1,000 W/m^2^·°C are 3.27 μm, 3.44 μm and 3.55 μm for the widths 15 μm, 30 μm and 45 μm, respectively. Thus, it can be seen that the change to high *h* medium decreased the deflections in the cantilevers. The decrease can be attributed to the lowering in maximum temperature observed in the same cantilevers when operated in the same environment (Figure 2). The relative decrease in maximum deflections is similar to the relative decrease in maximum temperatures shown in Figure 2. Therefore, the temperatures and the deflections are closely related.

**Figure 6.**
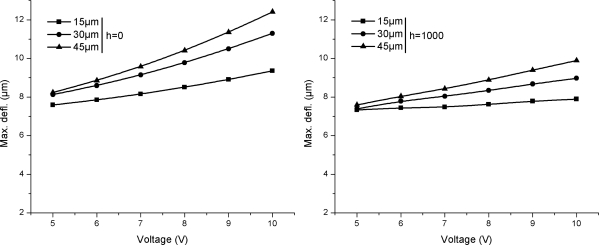
Maximum deflections in Si/SiO_2_ cantilevers.

Figure 6 shows the maximum deflections produced in Si/SiO_2_ cantilevers operated at *h* = 0 to *h* = 1,000 W/m^2^·°C. The maximum deflections for *h* = 0 are 9.36 μm, 11.30 μm and 12.41 μm, and for *h* = 1,000 W/m^2^·°C are 7.89 μm, 8.97 μm and 9.89 μm for the widths 15 μm, 30 μm and 45 μm, respectively. It is obvious in the figure that the change in cantilever substrate material from Si to SiO_2_ not only produced higher temperatures but also higher deflections. In fact, the deflection values observed in Si/SiO_2_ cantilevers are more than two times the Si/Si cantilevers. The increase in deflections can be attributed to the combined effect of higher temperatures and lower bending stiffness of Si/SiO_2_ cantilevers. The high temperatures in Si/SiO_2_ cantilever produce larger bimorph bending. In addition, the low elastic modulus of SiO_2_ reduces the bending stiffness of Si/SiO_2_ cantilevers. Therefore, high temperatures combined with low bending stiffness ultimately result in higher deflection in Si/SiO_2_ cantilevers. It can also be seen in Figure 6 that the relative decrease in deflections because of change in operating medium is more significant in case of Si/SiO_2_ cantilevers than in Si/Si cantilevers. This observation can be attributed to the relative larger temperature drop in Si/SiO_2_ cantilevers when operated in *h* = 1,000 W/m^2^·°C (Figure 3).

**Figure 7.**
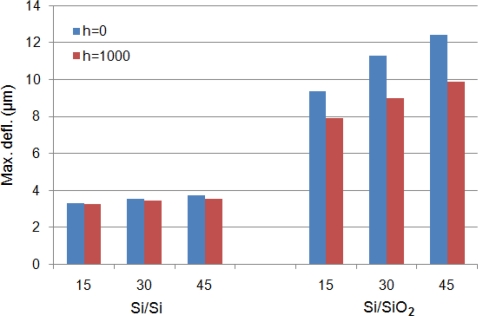
Comparison between maximum deflections in Si/Si and Si/SiO_2_ cantilevers at 10 V.

Figure 7 shows the comparison between maximum deflections observed in Si/Si and Si/SiO_2_ cantilever with different element widths for *h* = 0 and *h* = 1,000 W/m^2^·°C operated at 10 V. It is obvious in the figure that the change in cantilever substrate material from Si to SiO_2_ significantly increases the deflections, explained above. The deflection characteristics exhibited by Si/SiO_2_ cantilevers makes these especially useful in actuator applications. The relative decrease in the deflections in case of *h* = 1,000 W/m^2^·°C can be attributed to low temperatures in these cantilevers.

**Figure 8.**
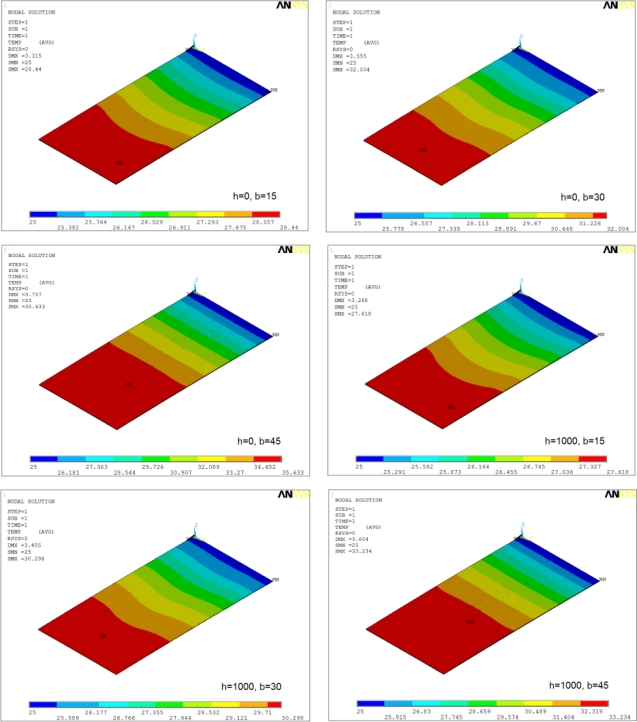
Temperature distribution in Si/Si cantilevers at 10 V.

Figure 8 shows the temperature distribution in Si/Si cantilevers for *h* = 0 and *h* = 1,000 W/m^2^·°C operated at 10 V. The distribution is one-dimensional and the temperature values increase along the cantilever length. The maximum temperatures occur at the tip of the cantilevers and the minimum at the cantilever base. The increase in element width increases the temperature and its domain in the cantilevers. In addition, the distribution remains unaffected by the change in operating medium.

**Figure 9.**
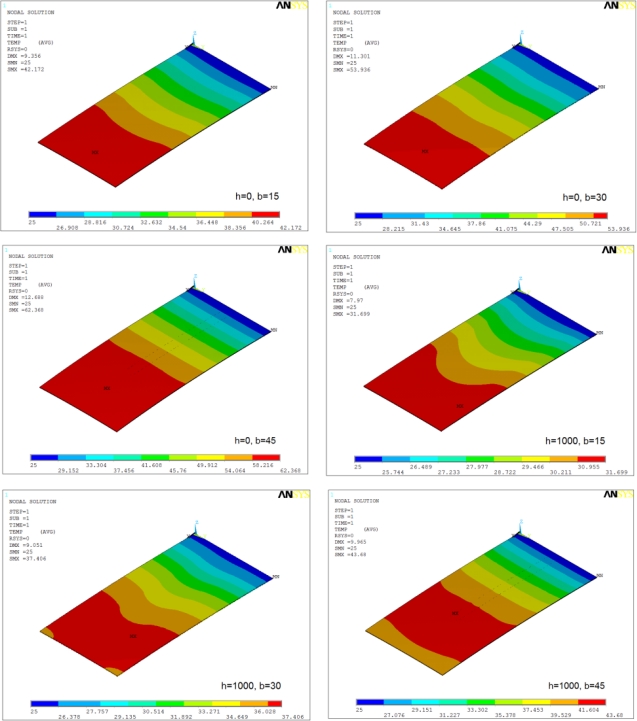
Temperature distribution in Si/SiO_2_ cantilevers at 10 V.

Figure 9 shows the temperature distribution in Si/SiO_2_ cantilevers for *h* = 0 and *h* = 1,000 W/m^2^·°C operated at 10 V. The distributions are similar to Si/Si cantilever, but the magnitudes are higher. The cantilever with wide element (*i.e.*, *b* = 45 μm) show discontinuous temperature distribution in case of *h* = 1,000 W/m^2^·°C. This behaviour can be attributed to the very high rate of heat loss from the tip region.

Now we discuss the self-heating associated with biosensor, AFM, hotplate and actuator application of doped microcantilevers. Self-heating is a major source of noise in piezoresistive microcantilevers used in biosensors and AFM applications [8,16]. The noise results from electrical sources like TCR and TCP and mechanical sources like bimorph bending and encapsulation stress. Zhou *et al*. [8] have shown that the resistance variation depends linearly on temperature and deflection. The noise can be reduced using appropriate temperature compensation mechanism or operating conditions. Chui *et al*. [17] proposed a novel temperature compensation mechanism for piezoresistive sensors based on the crystallographic orientation of the silicon cantilever microstructure. Thaysen *et al.* [16] proposed a symmetric balanced Wheatstone arrangement with a differential readout mechanism to reduce the noise. The thermal deflections can also be eliminated by depositing a passivated gold film on the bottom surface of the cantilever. A general technique to reduce thermal noise is to operate the cantilevers at low voltages and use advanced electronic amplifiers in measurements. Since self-heating depends on the square of the applied voltage, low voltages can help reduce significantly the temperatures produced, but low voltages will also make difficult the measurement of the voltage drop across the element.

A comparison between temperatures results presented in Figures 3 and 4 for self-heating produced in the doped cantilevers showed that Si/Si cantilevers have lower temperatures than Si/SiO_2_ cantilevers. It can also be observed in the figures that narrow element cantilevers produce less temperature than wide ones. In addition, the cantilevers operated in *h* = 1,000 W/m^2^·°C has lower temperature. Thus, the use of Si/Si cantilevers with narrow element and use in high *h* environment can help in reducing the noise generated by TCR and TCP. A similar comparison for deflections shown in Figures 5 and 6 indicates that Si/Si cantilevers have lower deflections than Si/SiO_2_ cantilevers. Further, the cantilevers with narrow element produce less deflection, thus reducing the noise produced by bimorph bending in the cantilever. In Si/Si cantilevers, there will be no encapsulation stress because the cantilever is a monolith structure made of silicon. The thermal expansion will be uniformly and continuously distributed in the entire structure. Therefore, we can conclude that Si/Si cantilevers with narrow elements are useful in reducing the thermal noise, and therefore are better suited for biosensor and AFM applications. In addition, the operating environment is also critical.

The purpose of a hotplate is to generate and maintain a localised temperature field on its surface. In this case, the element is a simple resistor and self-heating is necessary for proper functioning of the hotplate. Ability to achieve a rapid, high, and large uniform temperature field is primary requirement of a hotplate. Thermal deflections are generally neglected except for cases when self-sensing feature is also required. In case of microcantilever hotplates, the steady-state temperature field is normally achieved in ms [18]. A comparison between temperatures results presented in Figures 3 and 4 shows that Si/SiO_2_ cantilevers produce higher temperatures than Si/Si cantilevers, and therefore are more suitable for hotplate applications. Further, the cantilevers with wide element are desirable because of their high temperature features. Therefore, Si/SiO_2_ cantilevers with wide element are better for hotplates. Further, the decrease is cantilever tip temperature for *h* = 1,000 W/m^2^·°C cases, shown in temperature distribution results presented in Figure 9, suggest that for efficient performance the element should cover the entire length of the cantilever.

The cantilever tip deflections produced by the bimorph action can be used to useful effects like in nanolithography and AFM data storage. These applications often use the self-sensing and self-actuating feature of the doped cantilevers. Hence, the element is mostly a piezoresistor. Producing large cantilever tip deflection is a basic requirement. Large deflections produce large blocking force at the cantilever tip. A comparison between deflection results presented in Figures 6 and 7 shows that Si/SiO_2_ cantilevers are more suitable for actuators because of their ability to produce large deflections.

## 4. Conclusions

The present study employed numerical and analytical methods to study the effect of convection on self-heating in doped microcantilevers. The numerical results for maximum temperature produced in the cantilevers agreed well with the analytical model with the maximum deviation between the two being less than 6%. Results showed that temperature decreased rapidly with an increase in the convective coefficient of heat transfer. The temperatures produced in Si/SiO_2_ cantilevers were more than 1.5 times those of Si/Si. In addition, they were more affected by the operating environment. The thermal deflections were found to be closely related to the temperatures produced. The deflections in Si/SiO_2_ cantilevers were more than two times Si/Si. Finally, we found that although the temperature and deflections values are different, the temperature distribution in Si/Si and Si/SiO_2_ cantilevers are similar. A study on self-heating associated with doped cantilevers used in biosensor, AFM, hotplate and actuators found that Si/Si cantilevers are more suitable for biosensor and AFM applications, whereas, Si/SiO_2_ cantilevers for hotplate and actuator applications. Finally, Si/Si cantilevers are good for achieving self-actuating and self-sensing features.

## Figures and Tables

**Table 1.** Material properties of doped microcantilevers in μMKS units.

**Property**	**SiO_2_**	**Si**	**Au**
Thermal conductivity, *k* (pW/μm °C)	1.38 × 10^6^	150 × 10^6^	317 × 10^6^
Thermal expansion coefficient, *λ* (1/°C)	0.5 × 10^−6^	2.8 × 10^−6^	14.2 × 10^−6^
Specific heat, *c*_p_ (pJ/kg °C)	745 × 10^12^	712 × 10^12^	129 × 10^12^
Electrical resistivity, *ρ*_e_ (Tohm-μm)	----	1 × 10^−9^	----
Elastic modulus, *E* (MPa)	70 × 10^3^	160 × 10^3^	80 × 10^3^
Poisson’s ratio, *ν*	0.20	0.23	0.42
Mass density, *ρ* (kg/μm^3^)	2.22 × 10^−15^	2.32 × 10^−15^	19.3 × 10^−15^
